# Overview of the Trending Enteric Viruses and Their Pathogenesis in Intestinal Epithelial Cell Infection

**DOI:** 10.3390/biomedicines12122773

**Published:** 2024-12-05

**Authors:** Chi-Chong Chio, Jou-Chun Chien, Hio-Wai Chan, Hsing-I Huang

**Affiliations:** 1 Research Center for Emerging Viral Infections, College of Medicine, Chang Gung University, Kwei-Shan, Taoyuan 33302, Taiwan; d0801403@cgu.edu.tw (C.-C.C.); m1203109@cgu.edu.tw (J.-C.C.); d000018430@cgu.edu.tw (H.-W.C.); 2 Department of Medical Biotechnology and Laboratory Science, College of Medicine, Chang Gung University, Kwei-Shan, Taoyuan 33302, Taiwan; 3 Graduate Institute of Biomedical Sciences, College of Medicine, Chang Gung University, Kwei-Shan, Taoyuan 33302, Taiwan; 4 Department of Pediatrics, Linkou Chang Gung Memorial Hospital, Kwei-Shan, Taoyuan 33305, Taiwan

**Keywords:** enteric virus, intestinal epithelial cells, viral pathogenesis

## Abstract

Enteric virus infection is a major public health issue worldwide. Enteric viruses have become epidemic infectious diseases in several countries. Enteric viruses primarily infect the gastrointestinal tract and complete their life cycle in intestinal epithelial cells. These viruses are transmitted via the fecal–oral route through contaminated food, water, or person to person and cause similar common symptoms, including vomiting, abdominal pain, and diarrhea. Diarrheal disease is the third leading cause of death in children under five years of age, accounting for approximately 1.7 billion cases and 443,832 deaths annually in this age group. Additionally, some enteric viruses can invade other tissues, leading to severe conditions and even death. The pathogenic mechanisms of enteric viruses are also unclear. In this review, we organized the research on trending enteric virus infections, including rotavirus, norovirus, adenovirus, Enterovirus-A71, Coxsackievirus A6, and Echovirus 11. Furthermore, we discuss the gastrointestinal effects and pathogenic mechanisms of SARS-CoV-2 in intestinal epithelial cells, given the gastrointestinal symptoms observed during the COVID-19 pandemic. We conducted a literature review on their pathogenic mechanisms, which serves as a guide for formulating future treatment strategies for enteric virus infections.

## 1. Introduction

The gastrointestinal (GI) tract is the body’s first line of defense against ingested viruses. Constructed by a single layer of tightly packed intestinal epithelial cells (IECs), the apical surface of these epithelial cells faces the lumen side to resist pathogen invasion into the underlying lamina propria of the intestinal epithelium, acting as a natural physical barrier [1,2]. This intestinal barrier is critical in defensive processes, preventing pathogen invasion [3]. Enteric viruses are a group of viruses that cause intestinal diseases in humans via infection of the GI tract. The viruses are transmitted primarily via the fecal–oral route and enter the human body through contaminated water or food, initiating their replication in IECs. Symptoms caused by different enteric virus infections vary widely, ranging from mild symptoms such as vomiting, abdominal pain, and diarrhea to severe conditions such as encephalitis and pulmonary edema [4]. Generally, most enteric virus infections can be eradicated by the body’s immune system. However, infections in children can cause severe symptoms and even death, usually via dehydration, which is the leading cause of infant mortality (Table 1) [4,5,6].

Enteric viruses can also penetrate the epithelial cell physical barrier, which may exacerbate disease progression and increase the risk of viral spread to other tissues, potentially leading to systemic infections [7]. Therefore, understanding the impact of enteric virus infections on IECs is crucial for developing antiviral therapies and policies to prevent severe disease outcomes. In this review, we provide a brief overview of rotavirus, norovirus, enterovirus, and coronavirus, with an emphasis on their pathogenicity mechanisms after infecting IECs. In addition, we discuss potential routes through which these enteric viruses can cause systemic infection.

**Table 1:** An overview of enteric viruses and their pathogenesis in intestinal epithelial cell infections.

Potential Routes of Enteric Viruses That Causes Systemic Infection				
Enteric Virus Types	Infections and Mortality	Cellular and Molecular Mechanism of Infection	Types of Immune Cells Involved in the Infection	Refs.
Rotavirus (RV)	CNS disease: severity varies widely, from well tolerated to death. Hepatobiliary diseases: range from acute to chronic, high mortality (90%) due to obstruction of bile ducts. Respiratory illness, kidney disease, cardiovascular disease, autoimmune diseases: range from mild to death.	The NSP4 can act as a virporin, which may induce NO production, causing neurological damage. Virus can infect B cells and damage the biliary. Virus can infect and replicate in the liver, triggering inflammation.	Monocyte, B cell, lymphocyte, dendritic cell	[8,9,10,11]
Human Norovirus (HNoV)	Low-grade fever, muscle aches: usually mild.	Virus infection induces proinflammatory cytokine release and triggers innate and adaptive immune system activation.	Neutrophils, monocytes, T cell, macrophages	[12,13,14]
Enterovirus A71 (EV-A71)	Neurological disease, cardiopulmonary disease: range from acute to long-term sequelae.	Virus infection induces the production of cytokines, which may increase endothelial permeability, causing systemic inflammation,	Neutrophils, monocytes, T cell, macrophages, dendritic cell	[15,16,17]
Coxsackieviruses A6 (CV-A6)	CNS disease, myocardial damage: high mortality in CNS disease patients.	Virus infection triggers inflammation, apoptosis, and astrocyte activation, and blocks innate immune response, causing neural damage and systemic inflammation.	Neutrophils, monocytes, T cell	[18,19,20]
Echovirus 11 (Echo11)	Hepatitis, respiratory disease, meningitis, myocardial injury: usually asymptomatic or mild, but can be severe in infected neonates.	Virus infection induces inflammation and reduces Type 1 IFN production. Some reports show that virus infection may induce pyroptosis in cells.	Neutrophils, monocytes, T cell, macrophages	[21,22,23,24]
Severe acute respiratory syndrome coronavirus 2 (SARS-CoV-2)	Respiratory disease: range from mild to death, high mortality in patients with acute respiratory distress syndrome (ARDS). Cardiovascular disease: usually mild and self-limiting. Renal injury: usually mild. Liver disease: range from mild to severe. Neurological disease: range from mild to death. Immune system: range from mild to death, high mortality is associated with ARDS.	Dysregulates cytokine production or oxidative stress in respiratory tract, which may cause diffuse alveolar damage, airway inflammation, and remodeling. Reduces ACE2 expression, causing RAS system dysfunction and resulting in endothelial dysfunction, inflammation, and thrombosis. Virus infection causes macrophage and CD8^+^ T-lymphocyte infiltration, which is indicative of cytokine release syndrome in the kidney. Dysregulates the expression of tight junction and bile acid transporter proteins, causing defective tight junction formation and bile transportation. Neuronal apoptosis and neuroinflammation, which is indicative of the early stage of neurodegeneration. Virus infection causing a cytokine profile resembling sHLH, a hyperinflammatory syndrome characterized by hypercytokinemia.	Neutrophils, macrophages, dendritic cells, B cell, T cell	[25,26,27,28]

### 1.1. Rotavirus (RV)

Before 1970, the cause of infantile gastroenteritis was largely unknown. The first isolation of virus particles occurred in 1973 from the duodenal and fecal samples of a child with diarrhea. Because of its wheel-like appearance, the virus was named rotavirus (RV) [29]. RV is transmitted via the fecal–oral route, causing symptoms such as acute gastroenteritis and watery diarrhea in children under 5 years of age. RV infections are estimated to result in 260 million diarrhea cases worldwide each year, leading to approximately 130,000 deaths among children under five years of age. Additionally, RV accounts for approximately one-third of all severe diarrhea-related hospital admissions [30,31,32].

**Figure 1:**
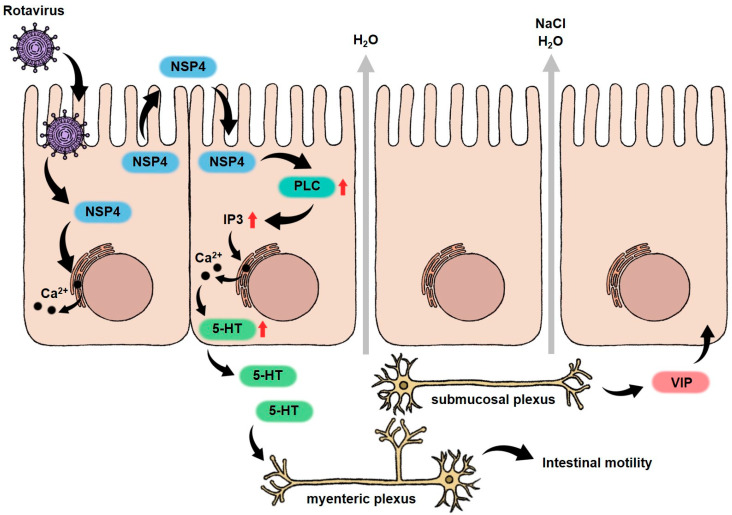
The mechanism of RV pathogenesis. RV produces NSP4 while infecting IECs. This protein promotes the transportation of Ca^2+^ from the ER to the cytoplasm, which causes Ca^2+^-dependent diarrhea. NSP4 can be secreted extracellularly, where it stimulates the expression of PLC in uninfected cells, increasing the production of IP3. This causes further release of Ca^2+^ from the ER into the cytoplasm. The resulting increase in cytoplasmic Ca^2+^ concentration disrupts tight junction integrity, leading to water influx into the intestinal lumen. Additionally, elevated cytoplasmic Ca^2+^ induces the secretion of 5-HT, which stimulates the myenteric plexus, enhancing intestinal motility. The stimulated myenteric plexus further activates the submucosal plexus to release VIP, increasing cAMP production in IECs. This leads to the secretion of NaCl and water into the intestinal lumen, ultimately causing diarrhea. RV, rotavirus; PLC, phospholipase C; IP3, inositol 1,4,5-trisphosphate; 5-HT, 5-hydroxytryptamine; and VIP, vasoactive intestinal peptide.

RVs belong to the *Reoviridae* family and are triple-layered particles: the layers include the outer capsid layer (VP4 and VP7), the middle capsid layer (VP6), and the inner capsid layer (VP2), which enclose a genome consisting of 11 segments of double-stranded RNA [33]. RV primarily infects enterocytes in the intestinal villi and impairs the absorptive function of epithelial cells [31]. RV nonstructural protein 4 (NSP4) has been identified as an enterotoxin. Intraperitoneal administration of NSP4 to mice results in diarrhea, demonstrating that NSP4 plays an important role in pathogenesis [34]. Hyser et al. reported that the hydrophobic domains of NSP4 function as viroporins, which cause Ca^2+^-dependent diarrhea [35]. RV-infected intestinal cells release NSP4, which stimulates uninfected cells to express phospholipase C, leading to the production of inositol 1,4,5-trisphosphate and an increase in the cytoplasmic Ca^2+^ concentration. Moreover, it prompts the enterochromaffin cells to secrete 5-hydroxytryptamine, activating the myenteric plexus and promoting intestinal motility. This, in turn, triggers nerve endings to release vasoactive intestinal peptides, causing crypt cells to release NaCl and water into the intestinal lumen, contributing to secretory diarrhea (Figure 1) [8,36,37,38].

In addition to the virus causing gastroenteritis, RV infection is also associated with type 1 diabetes, respiratory diseases, myocarditis, and various neurological diseases. Animal models have demonstrated that RV RNA can be detected in mesenteric lymph nodes, liver, kidneys, and other organs, indicating extraintestinal RV infections (Table 2) [39]. The most prevalent extraintestinal symptoms of RV infection are central nervous system (CNS) diseases, including seizures, encephalitis, cerebral white matter abnormalities, and cerebellitis [40]. The precise mechanisms by which RV invades the CNS remain unclear, and the causes of neurological diseases likely involve multiple pathways. Previous research has shown that the abnormal increase in intracellular Ca^2+^ levels induced by NSP4 promotes nitric oxide (NO) production, which causes damage to neurons [37,41]. Elevated NO levels in the cerebrospinal fluid (CSF) of patients with RV-induced seizures support this hypothesis [42]. Additionally, the immune response triggered by RV infection may contribute to neuronal damage. Yeom et al. reported that monocyte chemoattractant protein-1 (MCP-1) expression is elevated in patients with cerebral white matter abnormalities. Other inflammatory cytokines are also found at high levels in the CSF of patients with CNS complications, further contributing to neuronal damage [43,44].

**Table 2:** In vivo and in vitro studies to understand the pathogenesis of enteric viruses in intestinal epithelial cell infections.

Enteric Viruses Types	In Vivo Model/In Vitro Model	Mechanism of Pathogenesis	Results	Clinical Interpretations	Refs.
Rotavirus (RV)	In vivo: mice, pig, rabbit In vitro: human intestinal epithelium cell lines, human intestinal enteroids (HIEs)	Virus infection destroys the villus enterocytes and then decreases the absorption of sodium ions, water, and disaccharidases. The release of viral NSP4, which acts like an enterotoxin, increases the release of calcium ions and innervates intestinal motility.	Villous atrophy, malabsorption, enteric nervous system activated	Watery diarrhea, vomiting, gastroenteritis, and fever	[8,10,33,45,46]
Human Norovirus (HNoV)	In vivo: zebrafish, gnotobiotic pigs, gnotobiotic calves. In vitro: B cell, intestinal enteroids.	Human Norovirus (HNoV) infection leads to the shortening of microvilli, compromising the epithelial barrier. VP1 further promotes the production of AQP1, which increases intestinal barrier permeability.	Blunting of the villi, enlarged and pale mitochondria, intercellular edema, malabsorption	Watery diarrhea, nausea, vomiting, malaise, abdominal pain, gastroenteritis	[47,48,49,50]
Enterovirus A71 (EV-A71)	In vivo: mouse, non-human primates. In vitro: RD cell, Human intestinal epithelium cell lines, organoids, enteroids.	Virus infection triggers cell death or cell lysis to exit the cells.	Virus invades other tissues via circulatory system after breaking the intestinal epithelial barrier.	Hand, foot, and mouth disease (HFMD), neurologic complications, encephalitis, and paralysis	[1,51,52,53]
Coxsackieviruses A6 (CV-A6)	In vivo: mouse. In vitro: RD cell, Vero cell, KMB17 cell, HEK293A cell.	The KREMEN1 is the only known receptor for CV-A6. Virus infection may suppress interferon production and trigger cell death.	Altering host immune responses, Necroptosis	Herpangina, skin rash, vesiculobullous eruptions, desquamation, onychomadesis, and epididymitis	[20,54,55,56]
Echovirus 11 (Echo11)	In vivo: mouse. In vitro: RD cell, Vero cell, hepatocellular carcinoma cell lines, blood cells.	Virus utilizes CD55 and FcRn entering the cells and then activates the assembly of inflammasomes and triggers cell death.	Destroy epithelial barrier, Inflammation	Cough, rhinitis, hand, foot, and mouth disease (HFMD), myocarditis, aseptic meningitis, and rashes	[23,57,58]
Severe acute respiratory syndrome coronavirus 2 (SARS-CoV-2)	In vivo: mice, rabbits, hamster, bats, cats, dogs, and non-human primates. In vitro: 2D or 3D cultures of primary or immortalized cells and tissues.	Virus infects intestinal epithelial cells, causing cytokine and chemokine release and the downregulation of ACE2, which leads to disorder of the RAS system.	Acute intestinal inflammation, Declining the anti-inflammatory ability, Epithelial damage	Diarrhea, anorexia, vomiting, nausea, encephalitis, hepatitis, and inflammatory bowel disease	[59,60,61,62]

### 1.2. Norovirus (NoV)

Human NoV is currently a leading cause of acute gastroenteritis across all age groups, accounting for approximately 20% of diarrheal cases [63]. Following the first outbreak in Norwalk in 1968, NoV rapidly became the leading cause of viral gastroenteritis globally [4]. NoV infections often break out in winter and cause approximately 200,000 deaths annually [29]. NoV transmission occurs via person-to-person contact, contaminated food and water, and aerosol droplets. Owing to its transmission routes, NoV is a prevalent health-care-associated infection [64]. The typical symptoms of NoV infections include vomiting, abdominal cramps, the presence of mucus in the stool, and watery diarrhea. While healthy adults may experience mild illness or even asymptomatic infection, the virus can cause severe illness and has a high mortality rate in children, elderly individuals, and immunocompromised individuals [65].

NoV is a nonenveloped, positive-sense, single-stranded RNA virus belonging to the *Caliciviridae* family. After NoV infects the intestine, it causes significant damage to the intestinal villi, including broadening and blunting of the villi, crypt cell hyperplasia, and cytoplasmic vacuolization in the small bowel [66]. Furthermore, studies have reported that NoV patients with inflammatory bowel disease experience symptoms of hemorrhagic diarrhea [67]. Currently, the inability to culture human NoV (HNoV) in vitro has hindered the understanding of its mechanisms. The lack of an in vitro model for HNoV poses a significant challenge. While a few large animals exhibit mild diarrhea symptoms after being infected with HNoV, these limitations have hindered research on NoV [68]. However, recent studies have shown that human intestinal organoids support NoV infection and replication [69,70]. Additionally, in 2022, Ghosh et al. proposed a salivary gland cell model capable of NoV replication, which significantly overcomes the challenges associated with effectively amplifying NoV in vitro [71]. These organoid models have overcome the longstanding research bottlenecks associated with NoV, paving the way for future advancements in the study of NoV pathogenesis (Table 2).

NoV can be classified into six groups (GI to GVI) on the basis of VP1. Research data indicate that GII.4 variants have replaced previous NoV strains as the currently predominant strains [72,73]. The VP1 protein plays a crucial role in virus–cell receptor interactions. The P domain of NoV VP1 binds to histo-blood group antigens (HBGAs), facilitating viral attachment to cells [74]. HBGAs are present in the mucus layer and the surface of some cells, including enterocytes, making the GI tract the primary site of NoV infection [66]. Research on MNoV has identified CD300ld and CD300lf as receptors that facilitate viral entry into cells [41]. Both CD300ld and CD300lf are expressed in various myeloid cells, while CD300lf is also expressed in lymphoid cells and tuft cells within the intestinal epithelium (Table 3) [75]. However, these receptors are specific to MNoV and are not applicable to HNoV, whose receptors remain unidentified. However, whether diarrhea, transient malabsorption, and shortened microvilli caused by NoV infections directly affect IECs remains unknown [47].

NoV nonstructural proteins have been reported to be associated with the secretory activity of host cells. The NoV nonstructural protein p48 inhibits SNARE-mediated vesicular transport by interacting with vesicle-associated membrane protein-associated protein A (VAP-A), thereby disrupting the secretory capacity of host cells [76]. Another nonstructural protein, p22, blocks COPII-coated vesicles from transporting proteins from the ER to the Golgi, thereby inhibiting protein secretion [77]. This inhibition of host cell secretion increases the accumulation of vesicles within the cell, resulting in the assembly of the viral replication complex for NoV replication in host cells [78]. Most enteric pathogens similarly target secretory pathways and lead to electrolyte imbalances that result in diarrhea [79]. For example, VP1 has also been shown to promote the expression of the aquaporin 1 (AQP1) protein, increasing intestinal barrier permeability. This allows small molecules to pass through the intestinal barrier and potentially leads to diarrhea [80]. Research has also indicated that the recognition of the virus by Toll-like receptors (TLRs) triggers an inflammatory response, which may contribute to the development of irritable bowel syndrome and inflammatory bowel diseases in some patients [81,82,83].

**Table 3:** Overview of enteric viruses and their target receptors on intestinal cells.

Enteric Virus Types	Family	Genome	Structure Protein	Receptor on Intestinal Epithelial Cells	Refs.
Rotavirus (RV)	*Reoviridae*	11 segments of dsRNA	VP4	sialic acid, integrins, Hsc70	[38,84]
Human Norovirus (HNoV)	*Caliciviridae*	ssRNA (+), 7.5–7.7 kb	VP1	HBGA	[85]
Human adenovirus (HAdV)	*Adenoviridae*	dsDNA, 36 kb	fiber	CAR	[86]
			penton base	integrin	
Enterovirus A71 (EV-A71)	*Picornaviridae*	ssRNA, 7.4 kb	VP1	PSGL-1, SCARB2	[87]
Coxsackievirus A6 (CV-A6)				KREMEN1	[54,88]
Echovirus 11 (Echo11)			VP2 and VP3	CD55	[89,90]
			VP1	FcRn	
Coronavirus (CoV)	*Coronaviridae*	ssRNA, 27–32 kb	spike	ACE2	[91]

### 1.3. Adenovirus (AdV)

Adenovirus (AdV) is a nonenveloped, double-stranded DNA virus in the *Adenoviridae* family that is transmitted primarily through respiratory droplets or the fecal–oral route [92]. AdV infections can lead to a range of illnesses, including respiratory symptoms, pneumonia, fever, gastroenteritis, myocarditis, and conjunctivitis [93,94,95]. Common gastrointestinal symptoms include diarrhea, nausea, vomiting, and abdominal pain [94]. In children, AdV is a significant cause of severe diarrhea, whereas in immunocompromised patients, it can lead to serious complications such as meningoencephalitis, contributing to high morbidity and mortality in these populations [96,97,98,99]. Patients with AdV infection often exhibit intestinal epithelial cell damage, villus atrophy, and compensatory crypt hyperplasia, which results in malabsorption and fluid loss via the intestine [98,100].

AdV was first isolated from adenoid tissue in 1953 and, to date,, 51 serotypes and more than 100 genotypes of AdV have been identified. Humans are not the sole hosts for AdV; human AdVs (HAdVs) are classified into seven species, A to G [101,102]. Species A, F, and G target the gastrointestinal tract, primarily causing gastroenteritis and diarrhea. Among them, species F is the most common, with HAdV-F40 and HAdV-F41—collectively referred to as enteric adenoviruses—ranking as the second most common viral agents of gastroenteritis and diarrhea in children worldwide [102]. Species B, C, and E predominantly infect the respiratory tract, leading to pneumonia and acute respiratory infections, with species C also capable of causing hepatitis and pharyngitis. Species B and D can infect the eyes, resulting in keratoconjunctivitis, whereas species B is also associated with renal and urinary tract infections. Most studies have focused on the interactions between HAdV-C and host cells. However, it is HAdV-F that primarily causes gastroenteritis in children, leading to diarrhea and vomiting [103,104,105].

Currently known HAdV receptors include the Coxsackie and Adenovirus Receptor (CAR), desmoglein-2 (DSG2), CD46, polysialic acid, and GD1a (Table 3) [106]. Different HAdV types use distinct receptors for cell entry. CAR, which is broadly expressed on cell surfaces, serves as the primary receptor for most HAdVs, binding to the fiber knob proteins on their surface [106,107,108]. DSG2, a component of desmosomal junctions and an adhesion protein between epithelial cells, has a high affinity for the fiber proteins of HAdV-B types 3, 7, 11, and 14 [109]. Additionally, integrins (αvβ3 and αvβ5) are coreceptors that facilitate HAdV internalization. Unlike other HAdVs, HAdV-F encodes two distinct fiber proteins: a long fiber knob and a short fiber knob. The short fibers of HAdV-F increase the virus infectivity in intestinal cells [110,111]. The penton base proteins of HAdV-F40 and -F41 lack the arginine, glycine, and aspartic acid (RGD) sequences, which are typically used to bind integrin [103,112]. Instead, HAdV-F40 and HAdV-F41 have replaced this sequence with RGAD (Arg-Gly-Ala-Asp) and IGDD (Ile-Gly-Asp-Asp), respectively [113]. These unique characteristics may be associated with the intestinal tropism of HAdV-F, potentially influencing its preferential infection of gastrointestinal tissues.

Other receptors such as CD46, a member of the complement regulatory protein family, are involved in HAdV-16 and HAdV-35 (from the HAdV-B species) and HAdV-37 (from the HAdV-D species) attachment [94,114,115]. Notably, HAdV19 and 37 also employ integrins to infect human corneal cells via α2,3-sialic acid or CD46 [115,116]. However, receptors associated with HAdV-induced keratitis, such as α2,3-sialic acid and CD46, are widely expressed across various human cell types [117]. In polarized epithelial cells, CAR, DSG2, and integrins are expressed on the basolateral side, limiting HAdV particles from infecting intestinal cells via the apical side [118]. This distribution suggests that different HAdV types have a tissue tropism that enables infection beyond specific receptor-expressing cells or tissues, indicating a broader range of host tissue susceptibility [117].

The early genes of HAdV are translated into viral proteins, primarily E1, E2, E3, and E4, during the initial phase of infection, and are involved in viral replication and immune evasion. Among these, E1A regulates the cell cycle to the S phase, drives the transcription of E2, E3, and E4, and promotes viral proliferation [117,119,120]. Additionally, E1A has been shown to suppress NF-kB-dependent gene transcription and IFN-stimulated genes (ISGs) to reduce inflammation in the early stages of HAdV infection [121,122]. E1A is also known to induce the expression of p53, triggering apoptosis in host cells [123]. In contrast, the E1B protein, specifically the 55K product, binds to p53, blocking p53-dependent apoptosis [124]. Furthermore, Chahal et al. demonstrated that E1B can inhibit IFN antiviral activity, supporting HAdV replication [125]. In a lemur model, HAdV infection was observed to alter intestinal microbiota communities, notably increasing the abundance of the pathogenic genus Neisseria, which may lead to chronic immune stimulation in the gut and contribute to adenovirus-associated inflammatory bowel disease [98,126,127]. HAdV-induced meningoencephalitis is commonly observed in immunocompromised patients, such as those with AIDS or lymphoma. The presence of HAdV DNA in cerebrospinal fluid (CSF) confirms that HAdV can invade the central nervous system [128]. However, research on the pathogenic mechanisms of HAdV remains incomplete and requires further exploration.

### 1.4. Enteroviruses (EVs)

EVs belong to the genus *Enterovirus* within the *Picornaviridae* family. EVs are transmitted primarily through the fecal–oral route and respiratory droplets. The viruses are capable of infecting individuals of all ages, with the highest incidence and mortality rates observed in infants and young children [129]. Generally, most EV infections are asymptomatic or cause mild symptoms, such as hand, foot, and mouth disease (HFMD) and herpangina. However, for severe cases, virus infections can lead to aseptic meningitis, brainstem encephalitis, and even death [130,131]. On the basis of phylogenetic analysis of the VP1 protein, EVs are classified into four types: A, B, C, and D [130].

#### 1.4.1. Enterovirus A71 (EV-A71)

EV-A71 belongs to the Enterovirus A group. While it causes HFMD in young children, EV-A71 can also lead to neurological complications, and its antigens have been detected in patients who died from neurological diseases [132]. EV-A71 primarily infects human IECs and targets the GI epithelium early in its life cycle [15]. Additionally, an increasing number of studies suggest that EV-A71 infections may lead to pulmonary edema and death [133,134]. These findings indicate that EV-A71 can cross the intestinal barrier to infect various human organs and cause severe symptoms and even death. Some studies have shown that different EV-A71 varieties provide different levels of viremia, which promotes EV-A71 invasion of the brain, leading to neurological damage in mice [135]. These results indicate that EV-A71 might enter the bloodstream through a specific mechanism, further infecting various tissues and causing systemic infection, ultimately invading the CNS and leading to neurological complications. Nonetheless, there is insufficient research to elucidate the exact mechanisms of EV-A71 invasion into other tissues, and the mechanisms of EV-A71 neurotropism and systemic infections remain poorly understood.

EV-A71 utilizes various receptors, including P-selectin glycoprotein ligand-1 (PSGL-1), scavenger receptor class B member 2 (SCARB2), annexin II, sialic acid, nucleolin, and heparan sulfate, to facilitate infection (Table 3). Among these proteins, PSGL-1 and SCARB2 are essential for EV-A71 absorption and infection [136,137]. The viral capsid proteins VP1 and VP2 bind to receptors on the host cell membrane, initiating endocytosis to enter the cell and replicate [30]. In addition, Sun et al. discovered that the VP2 protein plays a significant role in EV-A71 neurotropism. In particular, a mutation at position 145 from V to I in VP2 enhances the ability of the virus to induce neurological symptoms via PSGL-1 in infected mice [134]. PSGL-1 is expressed on immune cells such as neutrophils, monocytes, lymphocytes, dendritic cells, and macrophages in the intestinal mucosal lymph nodes [138,139]. This widespread receptor suggests that EV-A71 can infect multiple tissues. Despite the understanding of the distribution of these receptors, the exact mechanism by which EV-A71 penetrates the intestinal barrier remains unknown.

**Figure 2:**
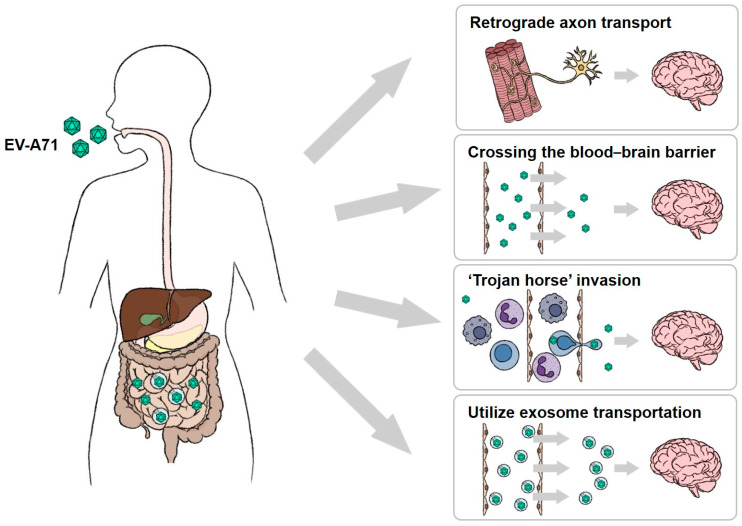
The potential route of EV-A71 invades the central neuron system. EV-A71 primarily infects and replicates in IECs. The generated EV-A71 exits from host cells by lytic and non-lytic processes and then invades the CNS via several potential pathways: (1) Retrograde axon transport: EV-A71 infects muscle cells and then enters spinal motor nerves, traveling retrogradely along axons to the CNS. (2) Crossing the BBB: EV-A71 circulates in the bloodstream and directly crosses the BBB to invade the CNS. (3) Trojan horse invasion: EV-A71 infects immune cells, which serve as carriers to transport the virus across the BBB into the CNS. (4) Utilize exosome transport: EV-A71 may be packaged within exosomes, which facilitate its crossing of the BBB and subsequent infection of neural cells. blood–brain barrier; EV-A71, Enterovirus A71; IECs, intestinal epithelial cells.

There are multiple hypotheses explaining EV-A71 transmission to other tissues (Figure 2). The virus is likely to first enter the small intestine and subsequently infect immune cells in the surrounding lymph nodes. This leads to viremia, which spreads the virus to various tissues via the transportation of immune cells [140,141]. Nevertheless, EV-A71 has been shown to increase vascular permeability through multiple mechanisms, which may help with virus transmission. For example, EV-A71 infection activates the NLRP3 inflammasome in macrophages and promotes the production of IL-1β to increase vascular permeability [142,143,144]. One study indicated that patients with brainstem encephalitis and pulmonary edema exhibit elevated serum and CSF levels of IL-6, TNF-α, IL-1β, and IFN-γ, suggesting that pulmonary edema may result from increased vascular permeability due to systemic inflammation [145]. Additionally, the VP1 protein of EV-A71 has been shown to reduce the expression of claudin-5 in brain endothelial cells while increasing vimentin expression, which leads to endothelial cell damage and increased vascular permeability [146]. Chen et al. proposed the possibility of retrograde axonal transport, where EV-A71 infects tonsillar epithelial cells and uses facial nerve fibers to invade the CNS [140,147]. Recent studies also suggest that EV-A71 may be transmitted distally via exosome secretion [148]. The virus does not activate apoptosis but uses exosomes to exit host cells via a nonlytic process to preserve the intestinal structure [149,150]. Gu et al. demonstrated that exosomes can cross the blood–brain barrier, facilitating EV-A71 infection in the CNS [151]. These studies suggest that EV-A71 may spread from the intestine to other tissues. However, research on EV-A71’s utilization of exosomes for exiting intestinal tissues remains preliminary. Additional evidence is essential to substantiate the hypothesis that EV-A71 employs exosomes as a mechanism to facilitate invasion into extraintestinal tissues.

#### 1.4.2. Coxsackievirus A6 (CV-A6)

CV-A6 and CV-A16 belong to Enterovirus species A, similar to EV-A71. Both CV-A16 and EV-A71 are primary pathogens that cause HFMD. CV-A6 was first isolated from HFMD patients in Finland in 2008 [152]. Since then, CV-A6-related HFMD cases have been reported globally, from Europe to South America and even Asia [153]. In China, the prevalence of CV-A6 infections has been reported to increase annually. Since the introduction of the EV-A71 vaccine in China and Taiwan, the incidence of EV-A71 infections has decreased, which makes CV-A6 the predominant cause of HFMD. In Shanghai, CV-A6 accounts for approximately 70% of HFMD cases, followed by CV-A16 at approximately 14% [154,155,156]. In recent years, CV-A6 has become a major global pathogen for HFMD, in contrast to EV-A71 [157]. Moreover, there has been as increase in adult HFMD cases associated with CV-A6 infections [29]. CV-A6 infections primarily result in herpangina and skin rash. Like EV-A71, CV-A6 can cause severe neurological complications and even death. CV-A6 infections often present unique clinical symptoms, such as widespread vesiculobullous eruptions, desquamation, onychomadesis, and epididymitis [158].

To date, most enterovirus research has focused on EV-A71 and CV-A16, with CV-A6 research being limited by the lack of established research models (Table 2) [18]. Although CV-A6 shares approximately 67% amino acid similarity with EV-A71 and CV-A16, it uses a different receptor for cell entry. The Kringle-containing transmembrane protein (KREMEN1) is currently the only known receptor for CV-A6 (Table 3) [54,88]. CV-A6 has been proven to infect 10-day-old mice intragastrically, resulting in intestinal edema. CV-A6 antigens have also been found in a variety of tissues, such as the lung, muscles, liver, and heart [159]. Sun et al. reported that CV-A6 can infect human glioma cells (U251 cells), and the virus-infected animals presented signs of brain edema and neuronal cell swelling [18]. These results indicate that CV-A6 can infect a variety of tissues. However, the mechanism by which CV-A6 travels from the GI tract to other tissues remains unknown.

#### 1.4.3. Echovirus 11 (Echo11)

Echo11 is a member of Enterovirus species B. The virus spreads primarily through fecal–oral and respiratory routes, but cases of vertical transmission from mother to infant during childbirth have also been reported [57]. In adults, Echo11 infections are typically mild or asymptomatic, and present mainly with respiratory symptoms such as cough and rhinitis [21]. However, Echo11 can also cause systemic symptoms, including HFMD, myocarditis, aseptic meningitis, and rashes [160]. Among the various echoviruses, Echo11 is associated with the highest rates of severe disease and mortality in neonates [161]. Neonates infected with Echo11 often exhibit symptoms of acute meningitis, hemorrhagic hepatitis, encephalitis, peripheral edema, hypoglycemia, thrombocytopenia, sepsis syndrome, and multiple organ dysfunction, which lead to high morbidity and mortality rates [22,57,162,163]. In 2018, Taiwan experienced an outbreak of Echo11, resulting in severe illness in eight neonates, seven of whom died [164]. In 2019, a nosocomial outbreak in Guangdong Province, China, led to the deaths of five infants with pneumonia [165]. In May 2023, France reported its first cases of Echo11 infection, which were subsequently observed across Europe [166]. Echo11 infection primarily leads to neurological complications and long-term sequelae in neonates, with an estimated one-third of infected neonates dying from these severe diseases [167]. Compared with patients infected with CVB5, another Enterovirus species B, Echo11 patients show more pronounced inflammatory responses and longer hospital stays [168].

CD55 and FcRn are the key receptors involved in Echo11 infection (Table 3). CD55 is present on most IECs and blood cells [169,170]. FcRn is expressed in intestinal enterocytes, where Echo11 completes its life cycle. FcRn has also been discovered in placental cells, hepatocytes, and vascular endothelial cells and is associated with the clinical symptoms observed in Echo11-infected patients [171]. Echo11 uses its VP2 and VP3 proteins to bind to CD55, and then the FcRn heavy chain interacts with the VP1 protein, facilitating the uncoating of Echo11 and the release of its genome in a neutral pH environment [30,89,90]. However, the roles of CD55 and FcRn in the infection process differ among various echoviruses [172]. Wells et al. demonstrated that Echo11 can infect enterocytes and that the virus disrupts the intestinal barrier structure. This finding provides evidence for the potential of Echo11 to spread from the intestine to other tissues [133]. Furthermore, Echo11 can activate the assembly of the NLRP3 inflammasome, leading to the production of IL-1β, which facilitates viral invasion of the brain and subsequent infection of neural cells [23]. Despite these insights, our understanding of Echo11 is still limited, and research into the pathogenic mechanisms of Echo11 is essential.

### 1.5. Coronavirus (CoV)

CoVs are a group of enveloped, positive, single-strand RNA viruses that belong to the family *Coronaviridae*, *Nidovirales* order. First recorded in 1912, coronavirus was an animal-related virus that was transmitted only between animal species. In the 1960s, the first human infection case was reported. The virus started to be transmitted to humans through the fecal–oral and respiratory routes and then became a zoonotic disease [173,174]. CoV can infect the respiratory tract, GI tract, and even the nervous system. In both humans and animals, the clinical symptoms include respiratory and GI complications [175]. Severe acute severe inflammatory responses or GI symptoms, such as cytokine storms, severe vomiting, and diarrhea, can potentially lead to patient death. Chronic inflammatory responses, such as encephalitis, hepatitis, and inflammatory bowel disease, may cause long-term multiorgan damage [174,176]. In recent years, viral receptors such as ACE2, TMPRSS2, and TMPRSS4, which are distributed in the intestine, have been strongly associated with coronavirus infections of intestinal cells (Table 3) [177]. Consequently, coronaviruses are increasingly recognized as significant enteric viruses.

Severe acute respiratory syndrome coronavirus 2 (SARS-CoV-2) first emerged in Wuhan city, mainland China, in 2020. According to WHO statistics, as of May 2023, there were 768 million confirmed cases globally, resulting in approximately 7 million deaths, drawing worldwide attention [63]. SARS-CoV-2 is an enveloped, single-stranded, positive-sense RNA virus that belongs to the *Betacoronavirus* family of *Coronaviridae*. The virus is transmitted primarily through fine aerosols expelled from the respiratory tract of infected individuals. Once these aerosols are inhaled, they infect the respiratory epithelium. Infected patients typically exhibit respiratory symptoms such as cough, fever, acute respiratory distress syndrome (ARDS), and pneumonia [178,179,180].

The receptor of SARS-CoV-2, angiotensin-converting enzyme 2 (ACE2), is present not only on the surface of respiratory epithelial cells but also on the gut, kidneys, and mature enterocytes. The coreceptors type II transmembrane serine protease (TMPRSS2) and TMPRSS4 are both highly expressed in the enterocytes of the GI tract, supporting the potential for SARS-CoV-2 to infect intestinal cells [74,181,182]. Zang et al. demonstrated that SARS-CoV-2 can infect IECs, providing evidence for GI symptoms such as vomiting and diarrhea in some infected patients, as well as the detection of viral RNA in feces [74]. Although SARS-CoV-2 is primarily a respiratory pathogen, even when viral RNA is no longer detectable in throat swabs, it can persist in feces for approximately seven days [63]. Furthermore, studies have identified viral envelope proteins in the intestinal tissues of COVID-19 patients, and live viruses have been isolated from fecal samples. In human intestinal organoid models, SARS-CoV-2 can replicate in enterocytes, suggesting the possibility of fecal–oral transmission (Table 2) [180,183,184,185,186].

The ACE2 receptor is downregulated after binding to the viral S protein, while an increase in proinflammatory cytokines affects the renin–angiotensin system. The SARS-CoV-2 spike protein activates the Ras-Raf-MEK-ERK pathway, increasing vascular permeability, which aligns with GI imaging findings of severe small intestine inflammation in COVID-19 patients. Increased vascular permeability facilitates viral entry into the bloodstream, increasing the risk of viral spread to other organs. In addition, increased permeability of epithelial cells allows microbes and toxins to enter the bloodstream, triggering an immune response and cytokine production, potentially leading to a “cytokine storm.” The cytokine storm induced by SARS-CoV-2 promotes platelet aggregation, causing microvascular thrombosis and leading to intestinal hypoxemia and cell necrosis [187,188,189,190].

## 2. Conclusions

Enteric viral infections are a major global public health threat. These viruses invade the human body primarily through the consumption of contaminated food or water, targeting intestinal cells upon entry into the body and causing villus atrophy and inflammation. The most common symptom of these viral infections is diarrhea. In certain cases, patients develop systemic infections, complicating treatment efforts. These infections account for approximately 5 billion cases each year, with mortality rates ranging from 15% to 30%, which is especially severe in low- and middle-income countries [191]. The rapid transmission and spread of enteric viruses place substantial pressure on health care systems in these regions.

The development of vaccines plays a crucial role in preventing gastrointestinal diseases caused by enteric viruses. Vaccines have already been developed for RV, NoV, and EV-A71, each of which has significant protective efficacy and has caused a reduction in mortality rates [192,193]. Although vaccines for certain enteric viruses are available, novel challenges continue to arise. For example, global RV vaccine coverage is approximately 50%, with considerably lower rates among low- and middle-income countries [193]. Some high-mortality countries are still evaluating factors such as vaccine efficacy and cost, which has led to limited RV vaccination in children and consequently high mortality rates in these regions. The expanded development and increased coverage of the EV-A71 vaccine resulted in a significant decrease in EV-A71 infections. However, this coincided with an increase in CV-A6, which has become the main cause of HFMD [154,155,156]. This underscores the importance of continued research on the pathogenic mechanisms of enteric viruses to support the design of more accessible vaccines and effective antiviral strategies.

Research efforts are currently limited by the lack of suitable infection models, particularly for CV-A6 and human NoV, which restricts our understanding of viral biology and the development of targeted therapeutics (Table 2) [69,194]. Future studies focusing on establishing robust infection models and elucidating the cellular targets of enteric viruses will be pivotal in overcoming these obstacles. These advancements are essential for developing innovative therapeutic strategies and ensuring global preparedness against enteric virus infections. In this review, we provide a comprehensive account of recent scientific discoveries and pathogenic mechanisms of enteric viruses, offering a foundation for developing therapies against these infections.

## Data Availability

Not applicable.
